# Confidence intervals for rate ratios between geographic units

**DOI:** 10.1186/s12942-016-0073-5

**Published:** 2016-12-15

**Authors:** Li Zhu, Linda W. Pickle, James B. Pearson

**Affiliations:** 1 Surveillance Research Program, Division of Cancer Control and Population Sciences, National Cancer Institute, National Institutes of Health, 9609 Medical Center Dr., Suite 4E346, Rockville, MD 20850 USA; 2 StatNet Consulting, LLC, Gaithersburg, MD 20882 USA

**Keywords:** Spatial autocorrelation, Rate ratio, Variance, Confidence intervals, Cancer statistics, Linked micromap plot

## Abstract

**Background:**

Ratios of age-adjusted rates between a set of geographic units and the overall area are of interest to the general public and to policy stakeholders. These ratios are correlated due to two reasons—the first being that each region is a component of the overall area and hence there is an overlap between them; and the second is that there is spatial autocorrelation between the regions. Existing methods in calculating the confidence intervals of rate ratios take into account the first source of correlation. This paper incorporates spatial autocorrelation, along with the correlation due to area overlap, into the rate ratio variance and confidence interval calculations.

**Results:**

The proposed method divides the rate ratio variances into three components, representing no correlation, overlap correlation, and spatial autocorrelation, respectively. Results applied to simulated and real cancer mortality and incidence data show that with increasing strength and scales in spatial autocorrelation, the proposed method leads to substantial improvements over the existing method. If the data do not show spatial autocorrelation, the proposed method performs as well as the existing method.

**Conclusions:**

The calculations are relatively easy to implement, and we recommend using this new method to calculate rate ratio confidence intervals in all cases.

## Background

Ratios of age-adjusted rates are of interest in public health research as a means of comparing rates in a set of geographic units with the rate in the overall area or in an area considered to be a “standard”. They are useful in providing information to the general public on the health condition of the community, and to policy stake-holders on program planning and priority setting. These rates (and ratios) between geographic units are correlated due to two reasons—the first being that each region is a component (sub-region) of the overall area; and the second often referred to as the “First Law of Geography” [1], i.e., everything is related to everything else, but near things are more related than distant things. The second source of correlation is called spatial autocorrelation.

Earlier work developed methods that estimated confidence intervals (CI) on age-adjusted rates and ratios of age-adjusted rates between non-overlapping regions [2] as well as between a sub-region and its parent region [3]. The latter took into account the correlation due to overlapping between the two regions, and showed that the *F* and the normal approximation were more efficient than the gamma approximation and the *F* interval [4]. Spatial autocorrelation is well known to be present in the distribution of diseases [5, 6] but has not yet been incorporated into rate ratio interval estimates published by the National Cancer Institute.

The Surveillance, Epidemiology, and End Results (SEER) Program of the National Cancer Institute [7] is an authoritative source of information on cancer incidence and survival in the United States. SEER currently collects and publishes cancer incidence and survival data from population-based cancer registries covering approximately 30% of the US population. Cancer statistics, including rate ratios, are released annually at the national, registry, state and/or county level via the statistical software SEER*Stat [8]. Rate ratios are provided but their interval estimates are computed assuming non-overlapping regions and no spatial autocorrelation.

In this paper, we present revised confidence intervals that take spatial autocorrelation into account and show that the interval coverage is more accurate and provides a higher statistical power. The proposed method considers both overlapping (non-spatial) and spatial autocorrelation. The three methods referred to throughout the paper are (1) the Tiwari method [3] that considers overlapping between a sub-region and its parent region; (2) the non-spatial method which is a special case in our proposal in that spatial autocorrelation is ignored and only overlap is accounted for; and (3) the spatial method that includes both overlap and spatial autocorrelation. Methods (1) and (2) are equivalent, only based on different probability distribution assumptions.

## Methods

### Age-adjusted rates

To study the health or disease status of a region, it is common to calculate the disease rate and compare it across geographic regions. A straightforward measure is the crude disease mortality (or incidence) rate which is calculated by dividing the total number of deaths (or new cases) in a given time period by the total number of people at risk during the same time period. A major problem with the crude rate is that it is an overall average rate that does not take into account possible confounding factors. The most common confounding factor for public health data is age, because many health conditions are age-related. Instead of the crude disease rate, the age-adjusted rate is usually calculated and reported to adjust for different age profiles between regions. Assuming that there are *I* geographic units and *J* age groups in the study area, and the data available are *D*_*ij*_, the number of deaths (or new cases), and *n*_*ij*_, the count of the population size from region *i* and age group *j*, then the age-specific rate, *R*_*ij*_, often expressed as number of cases per 100,000 people at risk, is calculated as

1 $$ R_{ij} = \frac{{D_{ij} }}{{n_{ij} }} \times 100{,}000 $$

Age-adjustment could be done internally, where the age-specific rate of the standard (or reference) population is weighted by the proportions of each age group in the study population [9]. A more common age-adjustment is called the direct method where the age-adjusted rate *R*_*i*_ of region *i* is calculated as

2 $$ \begin{aligned} R_{i} & = \sum\limits_{j = 1}^{J} {w_{j} } \frac{{D_{ij} }}{{n_{ij} }} \times 100{,}000 \\ & = \sum\limits_{j = 1}^{J} {w_{j} } R_{ij} \\ \end{aligned} $$

where *w*_*j*_ is the proportion of population size for age group *j* in the standard population and ∑_*j*=1_^*J*^ *w*_*j*_ = 1. Hence, the age-adjusted rate is the weighted average of age-specific rates, weighted by the standard population in order to minimize the effect of a difference in age distributions between regions. Let Ω denote the total region of interest, e.g., the entire U.S. Then the overall rate for Ω is computed by age adjusting after summing the number of deaths (numerator) and population (denominator) over all of the geographic regions, i.e.,

3 $$ \begin{aligned} R_{\varOmega } & = \sum\limits_{j = 1}^{J} {w_{j} } \frac{{\sum\nolimits_{i = 1}^{I} {D_{ij} } }}{{\sum\nolimits_{i = 1}^{I} {n_{ij} } }} \times 100{,}000 \\ & = \sum\limits_{j = 1}^{J} {w_{j} } R_{j} \\ \end{aligned} $$

For the rest of this paper, we use *R*_*i*_, *R*_*Ω*_ and *D*_*i*_, *D*_*Ω*_ to denote the random variables for the sub-regional and overall area age-adjusted rate and count respectively. The random variable of age-and region-specific rate, *r*_*ij*_, has expectation *λ*_*ij*_.

### Spatial autocorrelation

Suppose $\{ Z({\mathbf{s}}):{\mathbf{s}} \in {\mathbf{S}}\}$ represents a random process on surface ${\mathbf{S}} \in {\mathbf{R}}^{{\mathbf{2}}}$, and *Z*(*s*_1_), *Z*(*s*_2_), …, *Z*(*s*_*I*_) represents a partial realization of the random process. *Z*(·) is said to be second-order stationary if $E[Z({\mathbf{s}})] = \mu$ for all ${\mathbf{s}} \in {\mathbf{S}}$ (i.e., the mean of the process does not depend on location) and $Cov\left[ {Z\left( {s_{i} } \right), Z\left( {s_{{i^{{\prime }} }} } \right)} \right] = C\left( {s_{i} - s_{{i^{{\prime }} }} } \right)$ for all $s_{i} ,s_{i'} \in {\mathbf{S}}$. That is, the covariance function *C*(·), a measure of spatial correlation, depends only on the difference between locations *s*_*i*_ and $s_{{i^{{\prime }} }}$, not on the locations themselves. If $Var\left[ {Z\left( {s_{i} } \right) - Z\left( {s_{{i^{{\prime }} }} } \right)} \right] = 2\gamma \left( {s_{i} - s_{{i^{{\prime }} }} } \right)$, then *Z*(·) is said to be intrinsically stationary and the function 2*γ*(·) is called a variogram. The semivariogram, *γ*(·), has a value $r\left( {s_{i} - s_{{i^{{\prime }} }} } \right)$ which is a function of the difference between locations *s*_*i*_ and $s_{{i^{{\prime }} }}$ [10]. If, in addition, the covariance function *C*(·) does not depend on the direction between locations *s*_*i*_ and $s_{{i^{{\prime }} }}$, the process is called isotropic.

**Fig. 1:**
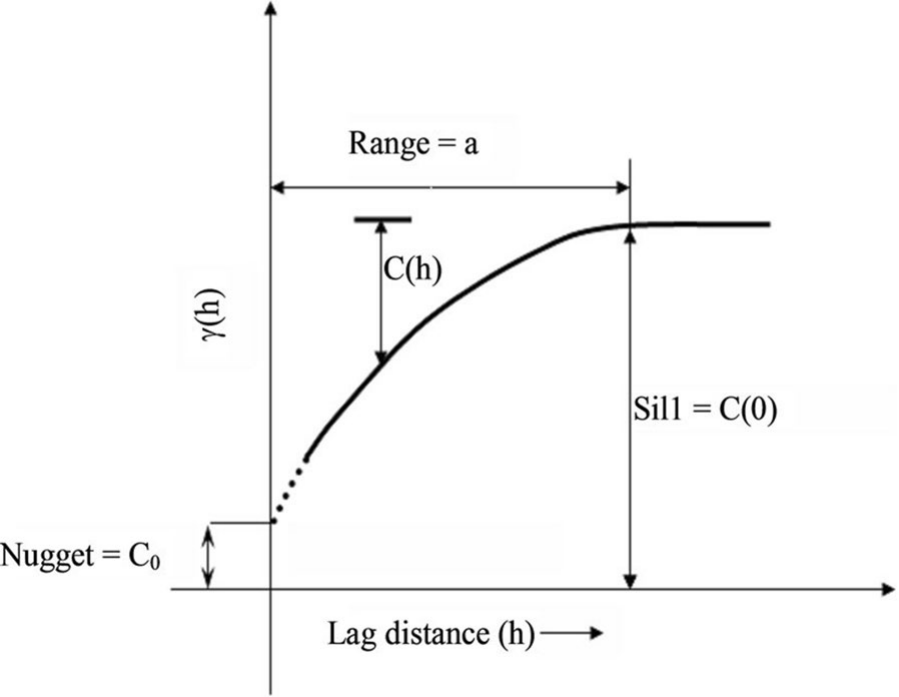
Illustrative semi-variogram plot. (credit: Samui and Sitharam [19])

For a stationary and isotropic spatial process, the semivariogram is a function of distance alone, i.e., $\gamma ({\mathbf{h}}) \equiv \gamma (||{\mathbf{h}}||)$ where $||{\mathbf{h}}||$ denotes the pairwise inter-point distance of vector ${\mathbf{h}}$. A plot of this function against separation distance, $||{\mathbf{h}}||$, conveys the spatial variability of the process (see Fig. 1). For a process with positive spatial autocorrelation, i.e., observations closer together are more alike than those further apart, the semivariogram value is non-decreasing with distance, indicating increasing variation with longer distance between two locations. Usually the semivariogram will approach a constant value (called the *sill*) at a large separation distance (called the *range*), beyond which the observations are considered spatially uncorrelated. The value of semivariogram at $||{\mathbf{h}}|| = 0$ is referred to as *nugget effect*, and it represents the variation between two observations that are fairly close together. If the *nugget effect* is positive (larger than 0), it may be due to measurement error or a spatially discontinuous process.

For a second-order stationary spatial process *Z*(·), semivariogram is related to the covariance function as

$$ \gamma ({\mathbf{h}}) = C({\mathbf{0}}) - C({\mathbf{h}}) $$

If $C({\mathbf{h}}) \to 0$ as $||{\mathbf{h}}|| \to \infty$, then $r({\mathbf{h}}) \to C({\mathbf{0}})$. So $C({\mathbf{0}})$ is the variance of $Z({\mathbf{s}})$ and the *sill* of the semivariogram. A *partial sill* is defined as the difference between the *sill* $C({\mathbf{0}})$ and the *nugget effect*, or $C({\mathbf{0}}) - r({\mathbf{0}})$. When we are comparing two spatial processes, it is useful to measure the correlation (spatial autocorrelation) instead of covariance. By definition, the spatial *correlogram* is

4 $$ \rho ({\mathbf{h}}) = \frac{{C({\mathbf{h}})}}{C(0)} = \frac{{C({\mathbf{0}}) - \gamma ({\mathbf{h}})}}{C(0)} $$

$\rho ({\mathbf{h}})$ is analogous to a typical correlation in that $|\rho ({\mathbf{h}})| \le 1$. When the distance between two locations exceeds the *range*, $r({\mathbf{h}}) = C({\mathbf{0}})$ and $\rho ({\mathbf{h}}) = 0$, i.e., there is no spatial autocorrelation.There are a few commonly used parametric semivariogram models, including spherical, exponential, Gaussian, and power models. A plot of the observed semivariogram of our lung cancer data suggested an exponential model, which is expressed as

5 $$ r({\mathbf{h}} ) = \left\{ {\begin{array}{*{20}l} {0,} \hfill &\quad {h = 0} \hfill \\ {c{}_{0} + c{}_{e}\left[ {1 - \exp ( - h/a{}_{e})} \right]} \hfill &\quad {h\text{ > }0} \hfill \\ \end{array} } \right. $$

where *c*_0_ is the nugget effect, *c*_*e*_ is the partial sill, and *c*_0_ + *c*_*e*_ is the sill. In this model, $r({\mathbf{h}})$ approaches the sill asymptotically and an effective range is defined as the distance at which the autocorrelogram is 0.05. Here, the effective range is 3*a*_*e*_ (see Fig. 1). Replacing $r({\mathbf{h}})$ in (4) with (5), the spatial correlogram becomes

6 $$ \rho ({\mathbf{h}}) = \left\{ {\begin{array}{*{20}l} 1 \hfill &\quad {h = 0} \hfill \\ {\frac{{c{}_{e}}}{{c{}_{0} + c{}_{e}}}\exp ( - h/a{}_{e})} \hfill &\quad {h\text{ > }0} \hfill \\ \end{array} } \right. $$

A larger proportion of partial sill to sill, $\frac{{c{}_{e}}}{{c{}_{0} + c{}_{e}}}$, and longer range, *a*_*e*_, mean stronger spatial autocorrelation.

### Variance calculation

Using notation similar to Tiwari et al. [2] where variances and confidence intervals of age-adjusted rates were derived, let ${\mathbf{r}} = ({\mathbf{r}}_{1} , \ldots ,{\mathbf{r}}_{\text{I}} )^{{\prime }}$ denote the vector of age-specific rates for the regions 1 through *I*, and each component $\text{r}_{\text{i}} = (r_{i1} , \ldots ,r_{iJ} )^{{\prime }}$ represent the rates for the *J* age groups in region *i*. Also let ${\bar{\mathbf{R}}} = (R_{1} , \ldots ,R_{I} ,R_{\varOmega } )^{{\prime }}$ denote the vector of age-adjusted rates for regions 1 through *I* and the overall age-adjusted rate *R*_*Ω*_. Tiwari et al. [3] derived confidence intervals (and therefore the relevant variances and covariances) for an age-adjusted rate and of the difference and ratio of two age-adjusted rates, specifically *R*_*i*_ and *R*_*Ω*_. as above. The calculation took into account the correlation due to the overlap between the sub-regions and the parent region. The derived 95% CI were shown to be more efficient than previously proposed methods [4]. However, both of these derivations ignored potential spatial autocorrelation among the area-specific rates. In this report, we will follow the development of Tiwari et al. [3] to derive the variance/covariance matrix for ln (*R*_*i*_/*R*_*Ω*_), the logarithm of the rate ratio for region *i* relative to the overall rate, adding spatial autocorrelation as necessary.

The age-adjusted rate vector is a linear combination of the age-specific rate vector,

$$ \overline{{\mathbf{R}}} = A{\mathbf{r}} $$

where

$$ A = \left[ {\begin{array}{*{20}c} {w^{{\prime }} } & 0 & \cdots & {} & 0 \\ 0 & {w^{{\prime }} } & 0 & \cdots & 0 \\ \vdots & {} & \ddots & \vdots & \vdots \\ {} & {} & {} & \ddots & 0 \\ 0 & \cdots & \cdots & 0 & {w^{{\prime }} } \\ {g_{1}^{{\prime }} } & {g_{2}^{{\prime }} } & \cdots & \cdots & {g_{I}^{{\prime }} } \\ \end{array} } \right],\quad g_{i}^{{\prime }} = \left( {\frac{{n_{i1} w_{1} }}{{\xi_{1} n}},\frac{{n_{i2} w_{2} }}{{\xi_{2} n}}, \ldots ,\frac{{n_{iJ} w_{J} }}{{\xi_{J} n}}} \right) $$

and *ξ*_*j*_ = *n*_*j*_/*n* =  ∑_*i*_ *n*_*ij*_/∑_*j*_ ∑_*i*_ *n*_*ij*_. So $Var(\overline{{\mathbf{R}}} ) = AVar({\mathbf{r}})A^{{\prime }}$; the dimensions of A are (*I* + 1) × (*IJ*) and the dimensions of the $Var({\mathbf{r}})$ variance/covariance matrix are (*IJ*) × (*IJ*), so that the dimensions of the $Var(\overline{{\mathbf{R}}} )$ matrix are (*I* + 1) × (*I* + 1). Using the Delta Method [11]:

$$ Var(\ln \overline{{\mathbf{R}}} ) \approx diag\left( {\frac{1}{{R_{1}^{2} }}, \ldots ,\frac{1}{{R_{I}^{2} }},\frac{1}{{R_{\varOmega }^{2} }}} \right)Var(\overline{{\mathbf{R}}} ) = diag\left( {\frac{1}{{R_{1}^{2} }}, \ldots ,\frac{1}{{R_{I}^{2} }},\frac{1}{{R_{\varOmega }^{2} }}} \right)AVar({\mathbf{r}})A^{{\prime }} $$

Our goal is to find the variance of ln(R_i_/R_Ω_), the logarithm of the ratio of the rate for area *i* to the overall rate:

$$ Var(\ln (R_{i} /R_{\varOmega } )) = (\begin{array}{*{20}l} 1 \hfill & { - 1} \hfill \\ \end{array} )Var\left( {\begin{array}{*{20}c} {\ln\,\,R_{i} } \\ {\ln\,\,R_{\varOmega } } \\ \end{array} } \right)\left( {\begin{array}{*{20}c} 1 \\ { - 1} \\ \end{array} } \right) = Var(\ln R_{i} ) + Var(\ln R_{\varOmega } ) - 2Cov(\ln R_{i} ,\ln R_{\varOmega } ) $$

Therefore, we need to compute $Var({\mathbf{r}})$, multiply by the factors in the above equation to estimate *Var*(*R*_*Ω*_), and then use the components of that result to compute the variance of the logarithm of the rate ratio.

Recall that age-place-specific rates ${\mathbf{r}} = (r_{11} ,r_{12} , \ldots ,r_{1J} ,r_{21} , \ldots ,r_{2J} , \ldots ,r_{I1} , \ldots ,r_{IJ} )^{{\prime }}$. Assuming that *D*_*ij*_ are spatially dependent Poisson random variables with means *n*_*ij*_ *λ*_*ij*_, we can write the variance of the age-place-specific rates as a matrix where the diagonal represents the variance of independent rates plus a matrix of off-diagonal terms representing the spatial autocorrelation. That is,

$$ \text{var} (r_{ij} ) = \text{var} \left( {\frac{{D_{ij} }}{{n{}_{ij}}}} \right) = \frac{{\text{var} (D_{ij} )}}{{n_{ij}^{2} }} = \frac{{\lambda_{ij} }}{{n{}_{ij}}},\;{\text{and}}\;{\text{so}} $$

$$ Var({\mathbf{r}}) = diag\left( {\frac{{\lambda_{11} }}{{n_{11} }}, \ldots ,\frac{{\lambda_{1J} }}{{n_{1J} }}, \ldots ,\frac{{\lambda_{I1} }}{{n_{I1} }}, \ldots ,\frac{{\lambda_{IJ} }}{{n_{IJ} }}} \right) + \text{cov} \left( {\frac{{D_{ij} }}{{n{}_{ij}}},\frac{{D_{{i^{{\prime }} j^{{\prime }} }} }}{{n{}_{{i^{{\prime }} j^{{\prime }} }}}}} \right) $$

where (*i*, *j*) ≠ (*i*′, *j*′). The Tiwari method assumed independence of *D*_*ij*_′s across both age and place, so that the 2nd (covariance) term in $Var({\mathbf{r}})$ above was a matrix of all zeroes. We will assume that the risk of disease is independent across age groups but the risk can be correlated among nearby places because of shared risk factors. That is, we assume independence across age but allow the possibility of spatial autocorrelation across places.

We will assume that the structure of spatial autocorrelation in our data follows the exponential form. Therefore $\text{cov} \left( {\frac{{D_{ij} }}{{n{}_{ij}}},\frac{{D_{{i^{{\prime }} j^{{\prime }} }} }}{{n{}_{{i^{{\prime }} j^{{\prime }} }}}}} \right) = \rho_{ii'} \sqrt {\text{var} \left( {\frac{{D_{ij} }}{{n{}_{ij}}}} \right)\text{var} \left( {\frac{{D_{{i^{{\prime }} j^{{\prime }} }} }}{{n{}_{{i^{{\prime }} j^{{\prime }} }}}}} \right)} = \rho_{ii'} \sqrt {\frac{{\lambda_{ij} }}{{n{}_{ij}}}\frac{{\lambda_{{i^{{\prime }} j^{{\prime }} }} }}{{n{}_{{i^{{\prime }} j^{{\prime }} }}}}}$ where $\rho_{{ii^{{\prime }} }}$, according to formula (6), represents the spatial autocorrelation between areas *i* and *i*′ with a form $\rho_{{ii^{{\prime }} }} = \frac{{c{}_{e}}}{{c{}_{0} + c{}_{e}}}\exp ( - h_{{ii^{{\prime }} }} /a{}_{e})$. Here $h_{{ii^{{\prime }} }}$ is the distance between the centroids of areas *i* and *i*′. In the case of positive spatial autocorrelation between two rates, $Var({\mathbf{r}})$ as calculated above will be the sum of two positive components. Therefore $Var({\mathbf{r}})$ will be a larger value than when assuming geographic independence, i.e., no spatial autocorrelation, as in the Tiwari method. Define a (*IJ*) × (*IJ*) matrix ${\mathbf{C}}$ as

$$ {\mathbf{C}} = \left[ {\begin{array}{*{20}c} 0 & {C_{12} } & \cdots & {C_{1I} } \\ {C_{12}^{{\prime }} } & 0 & \cdots & {C_{2I} } \\ \cdots & \cdots & \cdots & \cdots \\ {C_{1I}^{{\prime }} } & {C_{2I}^{{\prime }} } & \cdots & 0 \\ \end{array} } \right] $$

where $C_{{ii^{{\prime }} }} = \frac{{c{}_{e}}}{{c{}_{0} + c{}_{e}}}\exp ( - h/a{}_{e})\sqrt {\frac{{r_{ij} }}{{n{}_{ij}}}\frac{{r_{{i^{{\prime }} j^{{\prime }} }} }}{{n{}_{{i^{{\prime }} j^{{\prime }} }}}}}$ for *i* ≠ *i*′, and *C*_*ii*_ = 0, both are *J* × *J* matrix where the rows are indexed by *j* and the columns by *j*′. So

$$ Var(\ln (\overline{{\mathbf{R}}} ) = diag\left( {\frac{1}{{R_{1}^{2} }}, \ldots ,\frac{1}{{R_{I}^{2} }},\frac{1}{{R_{\varOmega }^{2} }}} \right)\left\{ {A\left( {diag\left( {\frac{{\lambda_{11} }}{{n_{11} }}, \ldots ,\frac{{\lambda_{1J} }}{{n_{1J} }}, \ldots ,\frac{{\lambda_{I1} }}{{n_{I1} }}, \ldots \frac{{\lambda_{IJ} }}{{n_{IJ} }}} \right)} \right)A^{{\prime }} + A{\mathbf{C}}A^{{\prime }} } \right\} $$

where **C** is the block matrix defined above. Multiplying the first term components yields

$$ diag\left( {\frac{1}{{R_{1}^{2} }}\sum\limits_{j} {\frac{{\lambda_{1j} w_{j}^{2} }}{{n_{1j} }},\frac{1}{{R_{2}^{2} }}\sum\limits_{j} {\frac{{\lambda_{2j} w_{j}^{2} }}{{n_{2j} }},} \ldots ,\frac{1}{{R_{I}^{2} }}\sum\limits_{j} {\frac{{\lambda_{Ij} w_{j}^{2} }}{{n_{Ij} }},} \frac{1}{{R_{\varOmega }^{2} }}\sum\limits_{i} {\sum\limits_{j} {\frac{{n_{ij} \lambda_{ij} w_{j}^{2} }}{{n^{2} \xi_{j}^{2} }}} } } } \right) $$

This matches the result of Tiwari et al. [2] Appendix A, for spatially independent places. Substituting for *ξ*_*j*_, the final term of this result can be rewritten as

$$ \frac{1}{{R_{\varOmega }^{2} }}\sum\limits_{i} {\sum\limits_{j} {\frac{{n_{ij} \lambda_{ij} w_{j}^{2} }}{{n^{2} \xi_{j}^{2} }} = \frac{1}{{R_{\varOmega }^{2} }}\sum\limits_{i} {\sum\limits_{j} {\frac{{n_{ij} \lambda_{ij} w_{j}^{2} }}{{n^{2} }}(I + 1)(I + 1)} } } } $$

Multiplying the components of the second term *A* **C** *A*′ results in a square matrix of dimension *I* + *1* that provides the additional variation and correlation due to spatial autocorrelation. Components of this matrix are

$$ \left\{ {\begin{array}{*{20}l} 0 \hfill & {for\;(i,i),\;i = 1,2, \ldots ,I} \hfill \\ {\frac{1}{{R_{i}^{2} }}\sum\limits_{{j^{{\prime }} }} {\sum\limits_{j} {w_{j} w_{{j^{{\prime }} }} \rho_{ii'} \sqrt {\frac{{\lambda_{{i^{{\prime }} j}} }}{{n{}_{{i^{{\prime }} j}}}}\frac{{\lambda_{{ij^{{\prime }} }} }}{{n{}_{{ij^{{\prime }} }}}}} } } } \hfill & {for\;(i,i^{{\prime }} \ne i),\;i = 1,2, \ldots ,I} \hfill \\ {\frac{1}{{R_{\varOmega }^{2} }}\sum\limits_{{i^{{\prime }} \ne i}} {\sum\limits_{{j^{{\prime }} }} {\sum\limits_{j} {\frac{{n_{{i^{{\prime }} j}} w_{j} w_{{j^{{\prime }} }} }}{{n_{j} }}\rho_{{ii^{{\prime }} }} \sqrt {\frac{{\lambda_{{i^{{\prime }} j}} }}{{n{}_{{i^{{\prime }} j}}}}\frac{{\lambda_{{ij^{{\prime }} }} }}{{n{}_{{ij^{{\prime }} }}}}} } } } } \hfill & {for\;(I + 1,i^{{\prime }} ),\;i^{{\prime }} = 1,2, \ldots ,I} \hfill \\ {\frac{1}{{R_{\varOmega }^{2} }}\sum\limits_{{i^{{\prime }} }} {\sum\limits_{{i \ne i^{{\prime }} }} {\sum\limits_{{j^{{\prime }} }} {\sum\limits_{j} {\frac{{n_{ij} n_{{i^{{\prime }} j^{{\prime }} }} w_{j} w_{{j^{{\prime }} }} }}{{n_{j} n_{{j^{{\prime }} }} }}\rho_{{ii^{{\prime }} }} \sqrt {\frac{{\lambda_{ij} }}{{n{}_{ij}}}\frac{{\lambda_{{i^{{\prime }} j^{{\prime }} }} }}{{n{}_{{i^{{\prime }} j^{{\prime }} }}}}} } } } } } \hfill &\quad {for\;(I + 1,I + 1)} \hfill \\ \end{array} } \right. $$

Combining relevant terms from the above results, we see that

7 $$ \begin{aligned} & Var(\ln (R_{i} /R_{\varOmega } )) = Var(\ln R_{i} ) + Var(\ln R_{\varOmega } ) - 2\text{cov} (\ln R_{i} ,\ln R_{\varOmega } ) \\ & \quad = \frac{{Var(R_{i} )}}{{R_{i}^{2} }} + \frac{{Var(R_{\varOmega } )}}{{R_{\varOmega }^{2} }} - 2\frac{{\text{cov} (R_{i} ,R_{\varOmega } )}}{{R_{i} R_{\varOmega } }} \\ \quad = \frac{1}{{R_{i}^{2} }}\sum\limits_{i} {\frac{{\lambda_{ij} w_{j}^{2} }}{{n_{ij} }}} + \frac{1}{{R_{\varOmega }^{2} }}\left( {\sum\limits_{i} {\sum\limits_{j} {\frac{{n_{ij} \lambda_{ij} w_{j}^{2} }}{{n_{j}^{2} }} + \sum\limits_{{i^{{\prime }} }} {\sum\limits_{{i \ne i^{{\prime }} }} {\sum\limits_{{j^{{\prime }} }} {\sum\limits_{j} {\frac{{n_{ij} n_{{i^{{\prime }} j^{{\prime }} }} w_{j} w_{{j^{{\prime }} }} }}{{n_{j} n_{{j^{{\prime }} }} }}\rho_{{ii^{{\prime }} }} \sqrt {\frac{{\lambda_{ij} \lambda_{{i^{{\prime }} j^{{\prime }} }} }}{{n_{ij} n_{{i^{{\prime }} j^{{\prime }} }} }}} } } } } } } } \right) \\ \quad \quad - 2\frac{1}{{R_{\varOmega } R_{i} }}\left( {\sum\limits_{j} {\frac{{w_{j}^{2} \lambda_{ij} }}{{n_{j} }} + \sum\limits_{{i \ne i^{{\prime }} }} {\sum\limits_{{j^{{\prime }} }} {\sum\limits_{j} {\frac{{n_{{i^{{\prime }} j}} w_{j} w_{{j^{{\prime }} }} }}{{n_{j} }}\rho_{ii'} \sqrt {\frac{{\lambda_{{i^{{\prime }} j}} \lambda_{{ij^{{\prime }} }} }}{{n_{{i^{{\prime }} j}} n_{{ij^{{\prime }} }} }}} } } } } } \right) \\ \end{aligned} $$

Formula (7) shows that the variance of a rate ratio is comprised of three components. If there were no correlation at all between the area *i* and standard rates, i.e., area *i* is not a sub-region of the standard and there is no spatial autocorrelation among the area rates, then the variance would simply be the first two terms in Eq. (7), $\frac{1}{{R_{i}^{2} }}\sum\nolimits_{i} {\frac{{\lambda_{ij} w_{j}^{2} }}{{n_{ij} }}} + \frac{1}{{R_{\varOmega }^{2} }}\sum\nolimits_{i} {\sum\nolimits_{j} {\frac{{n_{ij} \lambda_{ij} w_{j}^{2} }}{{n_{j}^{2} }}} }$, representing the case of no correlation. The variance due to overlap between region *i* and the overall region will be reduced by $2\frac{1}{{R_{\varOmega } R_{i} }}\sum\nolimits_{j} {\frac{{w_{j}^{2} \lambda_{ij} }}{{n_{j} }}}$, the fourth term of Eq. (7), representing the case of area overlap. The additional variance due to spatial autocorrelation is

8 $$ \frac{1}{{R_{\varOmega }^{2} }}\sum\limits_{{i^{{\prime }} }} {\sum\limits_{{i \ne i^{{\prime }} }} {\sum\limits_{{j^{{\prime }} }} {\sum\limits_{j} {\frac{{n_{ij} n_{{i^{{\prime }} j^{{\prime }} }} w_{j} w_{{j^{{\prime }} }} }}{{n_{j} n_{{j^{{\prime }} }} }}\rho_{{ii^{{\prime }} }} \sqrt {\frac{{\lambda_{ij} \lambda_{{i^{{\prime }} j^{{\prime }} }} }}{{n_{ij} n_{{i^{{\prime }} j^{{\prime }} }} }}} } } } } - 2\frac{1}{{R_{\varOmega } R_{i} }}\sum\limits_{{i \ne i^{{\prime }} }} {\sum\limits_{{j^{{\prime }} }} {\sum\limits_{j} {\frac{{n_{{i^{{\prime }} j}} w_{j} w_{{j^{{\prime }} }} }}{{n_{j} }}\rho_{{ii^{{\prime }} }} \sqrt {\frac{{\lambda_{{i^{{\prime }} j}} \lambda_{{ij^{{\prime }} }} }}{{n_{{i^{{\prime }} j}} n_{{ij^{{\prime }} }} }}} } } } $$

the third and fifth terms of Eq. (7). The first component in formula (8) is the additional variance in *Var*(ln *R*_*Ω*_) and the second component is the additional covariance in *Cov*(ln *R*_*i*_, ln *R*_*Ω*_) due to spatial autocorrelation. In most cases, adding spatial autocorrelation makes the additional variance (8) a negative value since the term representing −2 times the covariance dominates the sum of the two terms. When a sub-region accounts for a large proportion of the population in the parent region, the correlation between the sub-region and parent region is large because of a large overlap in the population, and so the additional covariance due to spatial correlation is relatively small. In that case, the first term in formula (8) dominates the overall value. Thus, although adding spatial autocorrelation will usually reduce the total variance (7), a high correlation between some sub-regions and the overall region can result in a larger variance for the rate ratio.

The full term in formula (7) is referred to as the spatial method, and subtracting the extra variance (8) due to spatial autocorrelation, we get the non-spatial version of the variance of the logarithm of rate ratio. To transform back to rate ratios, we apply the delta method again to get

9 $$ Var(R_{i} /R_{\varOmega } ) = (R_{i} /R_{\varOmega } )^{2} Var[\ln (R_{i} /R_{\varOmega } )] $$

All age-place-specific rate (*λ*_*ij*_) terms in the variance calculation will be approximated by the observed rates *R*_*ij*_.

## Results

To explore the impact of spatial autocorrelation in the confidence intervals of rate ratios, we will apply the methods with and without spatial autocorrelation to cancer incidence data from the SEER Program and cancer mortality data from the National Centers for Health Statistics [12]; both incidence and mortality data are available via the SEER*Stat software. The current SEER*Stat software provides age-adjusted rates with associated standard errors, confidence intervals, and between-geographic-area rate ratios with associated intervals and the p value (to test the rate ratio equals to 1). The rate ratio intervals are calculated using the Tiwari et al. [2] method (non-overlapping) but when rate ratio between two overlapping regions are requested, an alert message pops up that reads “The algorithms for the confidence intervals and the significance testing assume non-overlapping groups. Please use caution when interpreting these results.”

All age-adjusted rates in this paper are calculated using the 2000 US Standard Population [13] using the direct method, and the unit of the age-adjusted rates is per 100,000 people at risk. In addition to the real datasets, corresponding simulated rate ratios were created under the assumption that the logarithm of rate ratios comes from a spatial Gaussian process. The variation of the mean vector and variance–covariance structure will be described below. The simulation was implemented using the *spam* package [14] in *R* [15] with 10,000 realizations were created for each simulation.

### State-level data on different cancers

**Fig. 2:**
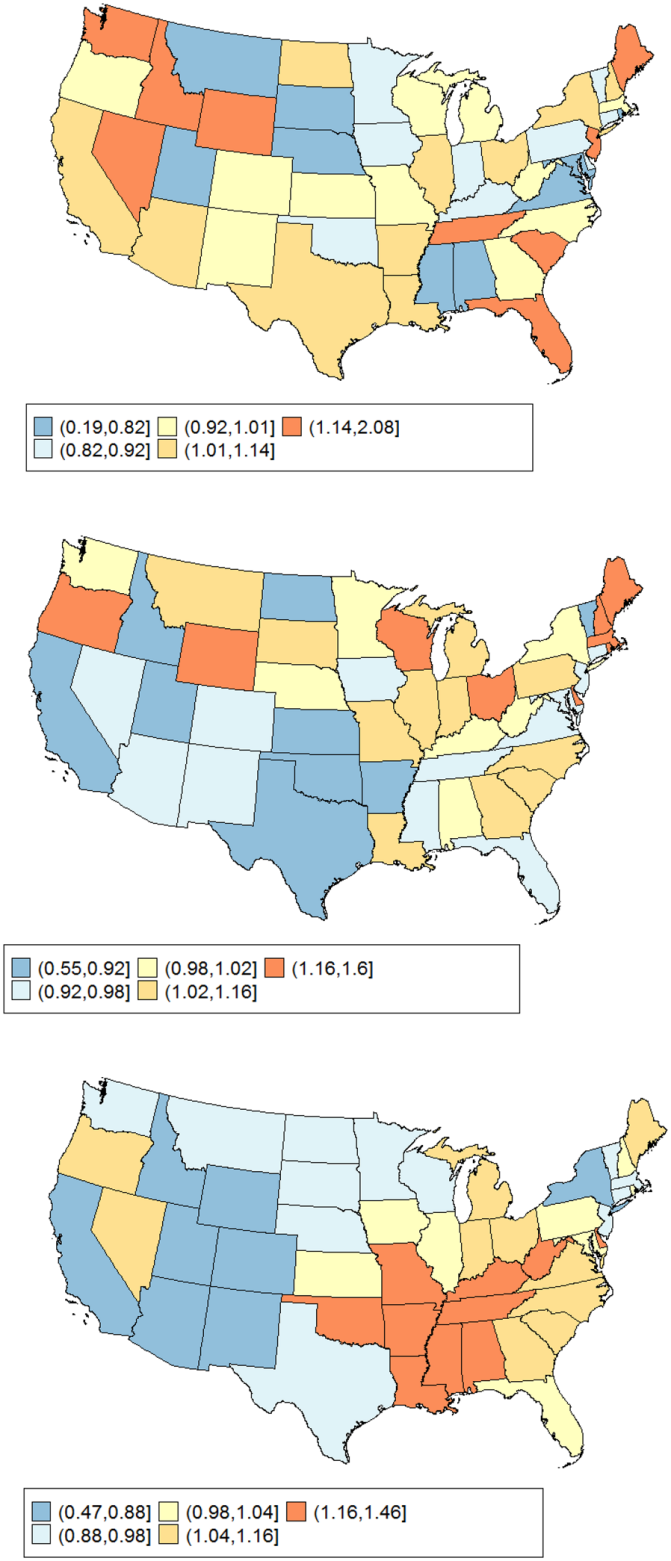
Rate ratio for tongue (*top*), esophagus (*middle*), and lung (*bottom*) cancer mortality in the US States, 2004

**Fig. 3:**
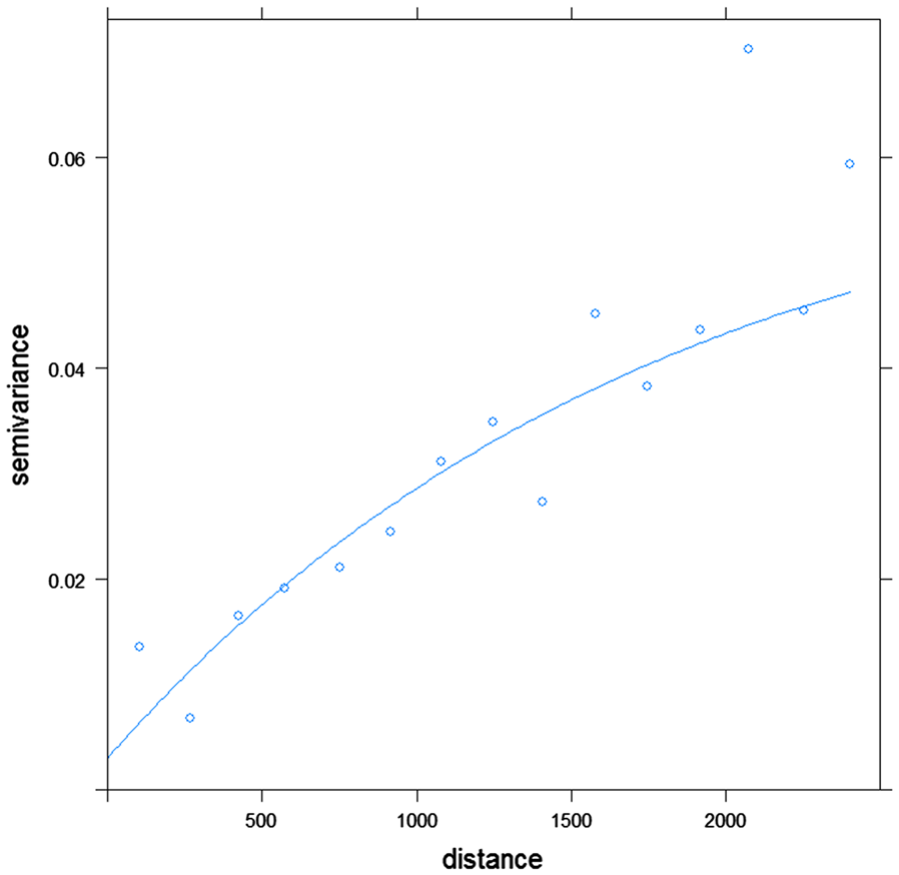
Empirical and observed semivariogram for lung cancer mortality in the US States, 2004

For rate ratios between a state and the US, the same data as in Tiwari et al. [3] were chosen to test the method developed above. Spe**c**ifically, age-adjusted mortality rates for tongue, esophagus, and lung cancer of the 50 states and the District of Columbia in year 2004 [16] were obtained and the ratios between the state rates and the US rate were calculated in SEER*Stat software. These three cancer sites are selected to represent the spectrum of cancer incidence, from rare cancer (age-adjusted mortality rate of 0.62 for tongue cancer in the US), to moderate (age-adjusted mortality rate of 4.35 for esophagus), and to common cancer (age-adjusted mortality rate of 53.30 for lung). The data also represent different levels of spatial autocorrelation in the state-level rate ratios (Fig. 2). Tongue cancer and esophagus cancer do not show a spatial pattern, but lung cancer has a clear spatial autocorrelation pattern. To estimate the variance–covariance structure between states, the observed semivariogram values (points in Fig. 3) for lung cancer mortality were modeled and plotted against the estimated values (curve in Fig. 3) using an exponential model. The exponential model produced an estimate of 0.06 for the *partial sill* and 0.065 for the *sill*, which implied that about 92% of the variation in the data can be attributed to spatial autocorrelation. The empirical range estimate was 1792 km, about one-third of the maximum state-to-state distance in the US. The semivariogram plot was cut off at half of the maximum distance, about 2500 km, since values beyond the range are uninformative. The parameter estimates from these models were used for the calculation of covariance matrix. Simulated data were created using the observed rate ratios as the mean and the estimated covariance matrix for each cancer site. Lengths of 95% CI and statistical power are compared between the Tiwari normal-based method, non-spatial and spatial method developed according to formulas (7)–(9). In the esophagus cancer data, the *partial sill* to *sill* ratio was 97% and the empirical range was only 98 km. The tongue cancer data had nugget effect = 0, and the empirical range was 104 km. Both situations indicate that states further than 100 km apart are basically considered spatially unrelated. We varied the strength and range in simulation studies, and the resulting data did not show any detectable spatial correlation for either cancer site, most likely due to the small number of cases.

**Fig. 4:**
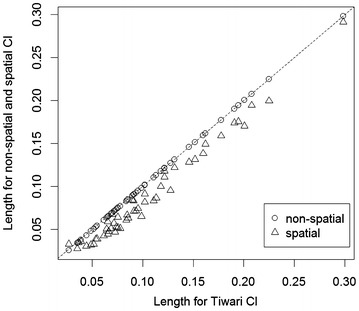
Length of 95% CI of State-to-US rate ratios, non-spatial and spatial method versus Tiwari method for simulated state-level lung cancer mortality data in the US

**Fig. 5:**
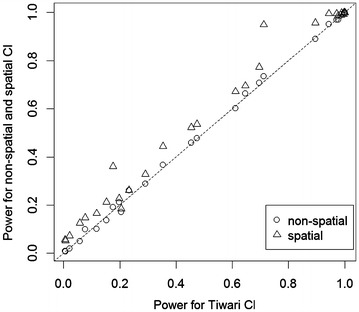
Statistical power of non-spatial and spatial method versus Tiwari method for simulated state-level lung cancer mortality data in the US

**Fig. 6:**
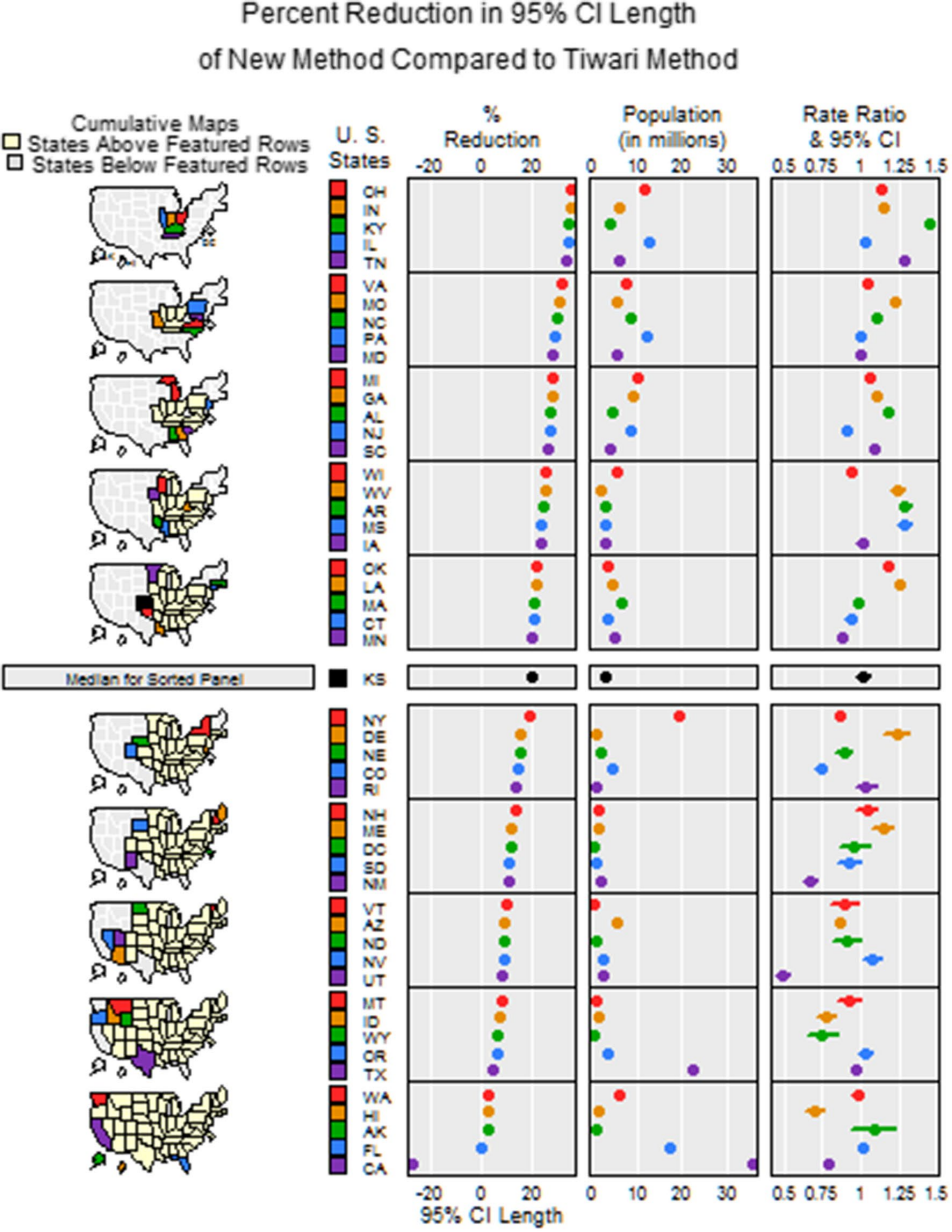
Linked micromap of percentage of reduction in the length of the 95% confidence intervals from the Tiwari method to the spatial method for lung cancer mortality rate ratios

Since lung cancer data show a clear spatial pattern, we focus on the results from lung cancer mortality State-to-US rate ratios in this sub-section. Figures 4 and 5 compare the Tiwari method, non-spatial and spatial methods in terms of the length of 95% CI for rate ratios and statistical power for the simulated state-level lung cancer data. It can be seen that the non-spatial method is very close to the Tiwari method in both plots. The spatial method is better than the Tiwari or non-spatial methods in that it provides shorter 95% CI and hence more accurate estimates, as well as higher statistical power. California is an anomaly in that adding correlation from both sources increases its variance estimate and resulting confidence interval length (see Fig. 6). The population in California accounts for about 12% of the US population, so the correlation between the state and US rates due to the overlap is much larger than the spatial autocorrelation. However, due to the large population size, the interval estimates for the rates (and rate ratios) were already very accurate and the statistical power was high, so the revised interval estimates and power were virtually unchanged.

A linked micromap plot [17] reveals the spatial pattern and associations between multiple variables simultaneously. To explore factors that contribute to the higher precision (and shorter confidence intervals) associated with the spatial method, we used a linked micromap plot (Fig. 6) to show the percent of length reduction in the 95% CI of lung cancer rate ratios from the Tiwari method to the spatial method in the US, expressed as percentages of the length of the Tiwari 95% CI for each state. The panels in the plot (from left to the right) are the maps of states with the highest to lowest % Reduction (from top to bottom row), the state names, the values of the % Reduction, the population size in each state and the estimated rate ratio with its 95% CI using the spatial method. The region with the highest % Reduction is in the mid-west, and expands to the east, west, and south, and finally to New England, Florida, and the Pacific West. Except for the few states with really large populations (e.g., New York, Texas, Florida, and California), there is a positive correlation between the population size and the % Reduction. In other words, the higher a state’s population size, the larger benefit it gains from the spatial method. Many of the larger states have such precise rate ratio estimates that the CI line is completely covered by the estimate’s dot. The only state that has a larger confidence interval using the spatial method is California, due to a relatively large proportion of the population of the state of the US total and hence a large overlap, as explained above. By adding the spatial correlation, the variance of the logarithm of the US rate, $\text{var} (\ln (R))$, increases from 6.4E−6 to 1.0E−4, a 15-fold change.

For esophagus and tongue cancer data, spatial autocorrelation is weak and the advantage of the spatial method is minimal. The results of the three measures are very close across the Tiwari, non-spatial, and spatial methods for esophagus and tongue cancer (data not shown).

### State-level data with varying autocorrelation strength and scale

To further explore the impact of spatial autocorrelation on the confidence intervals of rate ratios, we applied the competing methods to a set of simulated state-level data. The observed rate ratios of 2004 lung cancer mortality were taken as the mean, and the variance–covariance matrix was set with a variety of autocorrelation strength and scale. As shown in formula (6), for an exponential semivariogram model, spatial autocorrelation depends on the proportion of partial sill to sill, $\frac{{c{}_{e}}}{{c{}_{0} + c{}_{e}}}$, as well as the range, *a*_*e*_. The partial sill to sill proportion ranges between 0 and 1, with a higher value representing stronger spatial autocorrelation. The range is the distance between two regions beyond which the observations are considered spatially uncorrelated. Of the 50 US states and District of Columbia, the pairwise distance ranges from the minimum at 25.7 km (between District of Columbia and Maryland) to the median at 1608.0 km (between Florida and Oklahoma), and the maximum at 4984.0 km (between Maine and Hawaii). We set the range *a*_*e*_ in formula (6) at three different values, 500, 1700, and 4000 km, to represent local, regional, and global scale of spatial autocorrelation. The partial sill to sill proportion, $\frac{{c{}_{e}}}{{c{}_{0} + c{}_{e}}}$, was set at 0.1, 0.5, and 0.9, to represent weak, moderate, and strong spatial autocorrelation. A total of nine simulated datasets were created, and percent of length reduction from non-spatial method to spatial method was calculated for each state and averaged across the whole US.

Since the non-spatial method turns out to be very close to the Tiwari method, this section only compares the spatial method to the non-spatial method. Table 1 presents the percent of length reduction for the 95% CI from the non-spatial method to the spatial method, averaged across the 50 US states and District of Columbia, for varying scale and strength of spatial autocorrelation. At the local scale, when spatial autocorrelation strength increases from weak to strong, on average, the spatial method reduces the length of 95% CI by 0.48–4.5%, which is minimal. At the regional scale, the range of the semivariogram is 1700 km, which is about the average pairwise distance between US states. In other words, at the regional spatial autocorrelation scale, every state is related to about half of all other states. When the strength of the correlation increases from weak to strong, the spatial methods reduces the length of 95% CI by 1.7–17.7%. If the autocorrelation is on a global scale when every state is related to all other state, spatial methods reduces the length of 95% CI by 2.7–29.3%.

**Table 1:** Percent of length reduction for 95% CI for rate ratios, from non-spatial method to spatial method with varying spatial autocorrelation scale (measured by range in semivariogram) and strength (measured by partial sill to sill proportion in semivariogram)

	Scale of spatial autocorrelation measured by range (km)		
	500 (local)	1700 (regional)	4000 (global)
Strength of spatial autocorrelation			
0.1 (weak)	(0, 0.004, 0.095) 0.48	(0.005, 0.039, 0.098) 1.7	(0.029, 0.067, 0.099) 2.7
0.5 (moderate)	(0, 0.02, 0.47) 2.4	(0.027, 0.19, 0.49) 9.2	(0.14, 0.33, 0.50) 14.6
0.9 (strong)	(0, 0.036, 0.85) 4.5	(0.048, 0.35, 0.89) 17.7	(0.26, 0.60, 0.89) 29.3

Numbers in parentheses are the minimum (between Maine and Hawaii), median (between Florida and Oklahoma), and maximum (between Maryland and District of Columbia) pairwise correlation coefficients between regions

### County-level data

**Fig. 7:**
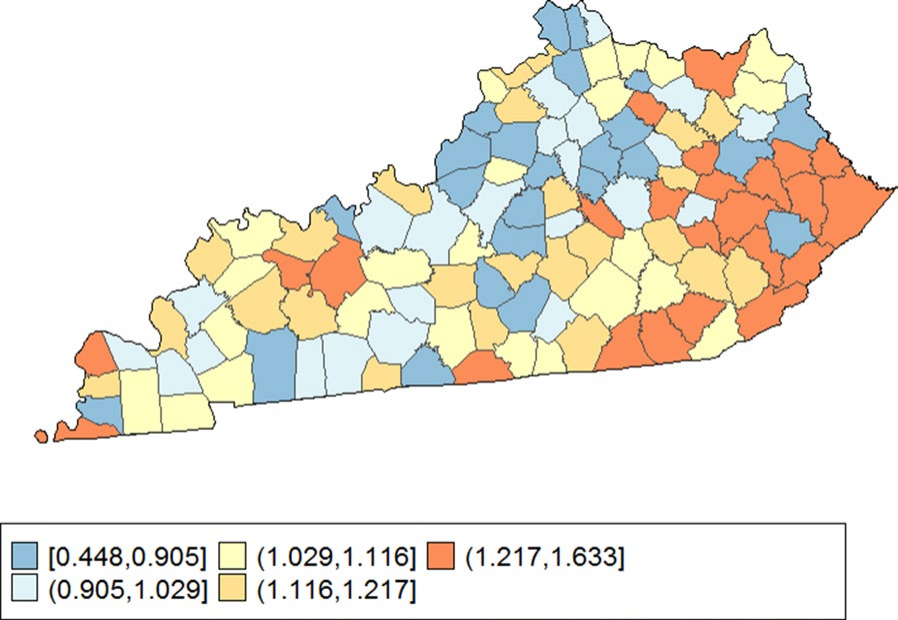
County-to-State ratio of age-adjusted rates for male lung cancer incidence in Kentucky, 2006–2010

The state of Kentucky has the highest cancer rates for both incidence and mortality. Cigarette smoking prevalence is high and cancer screening rate is low, especially in the southeast area of the state which is part of the Central Appalachia region [18]. We calculated the age-adjusted incidence rates of lung cancer for males for the 5-year period between 2006 and 2010. The state rate is 129.94 per 100,000 and the rates vary considerably among the 120 counties, from 57.01 to 207.21, resulting in the county-to-state rate ratios between 0.45 and 1.63 (Fig. 7). There is also a spatial pattern, with higher rates in the southeast mountain area, and lower rates in the north and central areas.

A simulation study was performed based on the Kentucky county-level male lung cancer incidence rates. The simulation study serves two purposes. First, we would like to establish the relationship among the three interwoven factors—county to state rate ratios, county population size, and statistical power in testing the hypothesis of rate ratios equal to 1. Then we would compare the non-spatial and spatial methods. To serve the first purpose, we simulated a set of county rates (according to the population size of each county in Kentucky) with the county-to-state rate ratios ranging between 0.2 and 2.0, with an increment of 0.05. This range is broader than that in a realistic situation and should provide a complete picture of the multi-dimensional relationship between rates, rate ratios, population size, and power. However, this is a hypothetical scenario since it is impossible to observe that all counties have the same rate ratio at a certain fixed level, so the analysis in this scenario only helps with understanding the impact of rate ratio and population size on the statistical power. It does not estimate how much improvement the spatial method will provide in a real world situation. To serve the second purpose, we created the simulated data assuming the hypothesized mean rate ratios and the variance–covariance structure were the same as the observed values. Then we compare the statistical power and coverage probability of the 95% CI between the non-spatial and spatial method.

Table 2 lists the statistical power and coverage probability of the non-spatial and spatial methods for the simulated Kentucky incidence data at a few hypothetical fixed rate ratios. The power of both non-spatial and spatial methods decreases from both small and large rate ratios toward 1.0, and power of the spatial method is consistently higher than that of the non-spatial method. The power increase is the largest when the county to state rate ratio is close to 1.0, and diminishes when the rate ratio turns further away from 1.0. For cancer data with a similar level of rates, the county to state rate ratio needs to be smaller than 0.7 or greater than 1.4 to reach a statistical power of 80% (data not shown). The coverage probability of the spatial method is very close to the nominal value of 95%, consistently better than of non-spatial method across all rate ratio values, which is around 97%.

**Table 2:** Power and coverage probability of the non-spatial and spatial methods in the simulation study for Kentucky county-level male lung cancer incidence data

	Power		Coverage probability	
	Non-spatial (%)	Spatial (%)	Non-spatial (%)	Spatial (%)
Simulation under hypothetical fixed rate ratios				
0.50	98.7	98.9	96.7	95.0
0.75	65.5	71.0	96.7	95.0
1.0	3.23	4.98	96.8	95.0
1.25	48.7	55.3	96.7	94.9
1.50	85.7	88.5	96.8	95.0
1.75	96.3	97.0	96.8	95.0

**Fig. 8:**
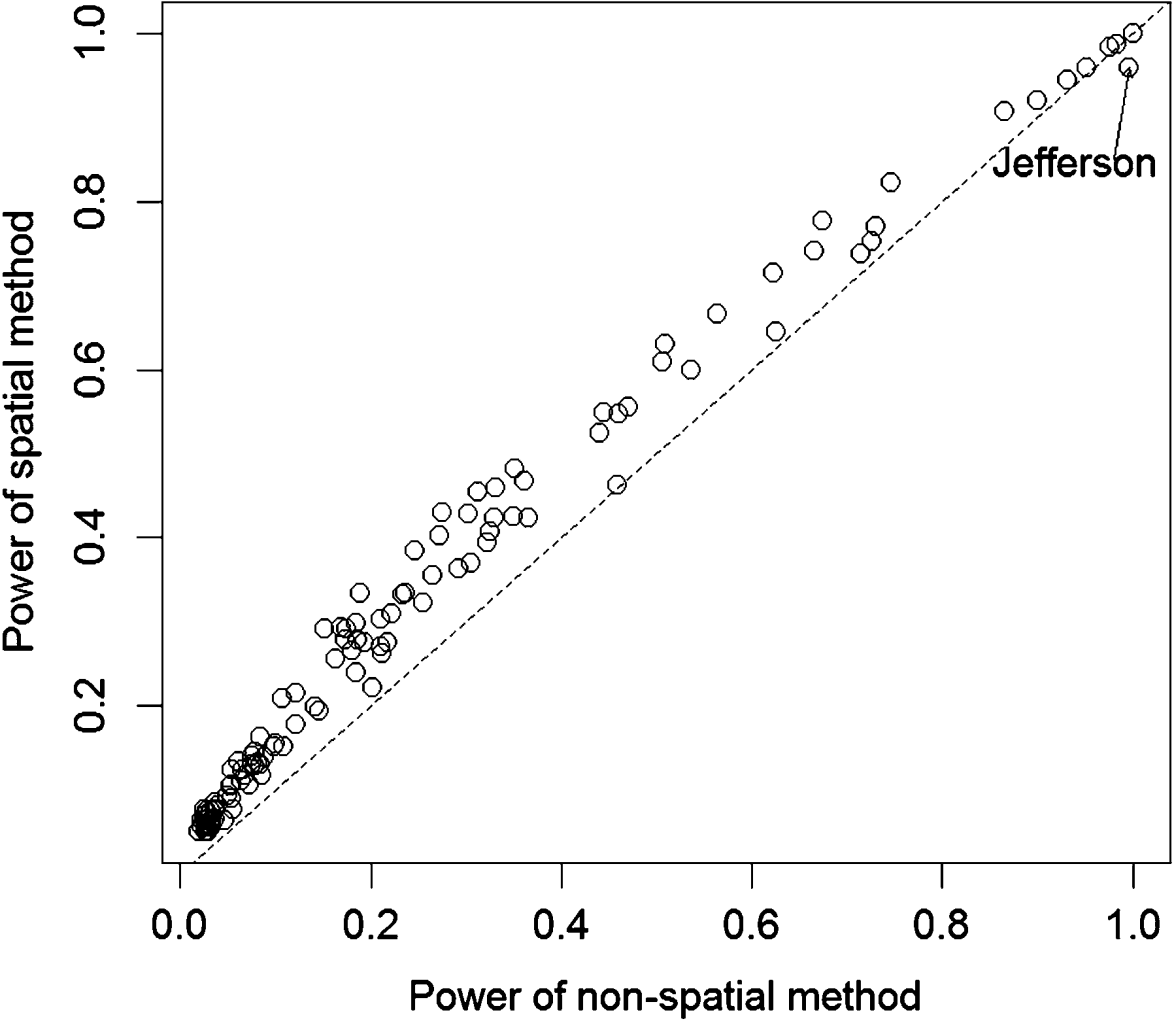
Statistical power of spatial method versus non-spatial method for simulated county level male lung cancer incidence data in Kentucky

**Fig. 9:**
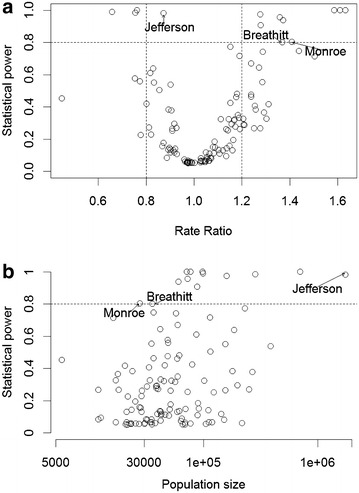
Statistical power of spatial method versus rate ratio (**a**) or population size (**b**) for simulated county level male lung cancer incidence data in Kentucky

Figure 8 compares the statistical power of the non-spatial and spatial methods for the same simulated data assuming the hypothesized mean rate ratios and the variance–covariance structure is the same as the observed values. It is clear that the spatial method has a higher power than the non-spatial method in all but Jefferson County, which accounts for about one-fifth of the population in Kentucky. The coverage probability of the spatial method is consistently better than that of the non-spatial method (not shown). Figure 9 reveals the relationship between statistical power vs rate ratio and population size in the simulated situation for the spatial method. Power is smallest when the rate ratio is close to 1.0, and increases as the rate ratio is further from 1.0. The counties with high power (greater than 0.8) have large populations (>70,000) and the rate ratios are either <0.8 or >1.2. Jefferson County’s population is the highest in the state, with a 5-year total of 1.76 million, which supports a high statistical power to detect its rate ratio of 0.87 as significantly different from 1.0. Two smaller counties (Monroe County with a 5-year population size of 27,000 and Breathitt County with a 5-year population size of 35,000) have rate ratios close to 1.4; their high rate ratios, rather than population size, drive their statistical power higher.

## Discussion

Presenting confidence intervals along with point estimates provides a measure of uncertainty of the estimate. Failure to include spatial autocorrelation when it is present can lead to errors in the estimates and subsequent inferential errors. We have extended a previous method of calculating confidence intervals for the rate ratio of a sub-region to the full region to include spatial autocorrelation. Tiwari et al. [3] proposed a method to compute a 95% confidence interval around the ratio of an area-specific rate to the rate for a larger area which includes that area. This method accounts for correlation between the single area rate and that of the larger area, but does not account for likely spatial autocorrelation between rates of neighboring areas. Our method includes components of total variance due to the standard distributional assumption, the correlation between the single area rate and the larger area rate, and spatial autocorrelation among the areas.

The rate ratio is a common measure of the relative ranking of areas, e.g., counties within a state, and is easily computed. The earlier non-overlapping method of Tiwari et al. [2] is now implemented in SEER*Stat, the National Cancer Institute’s popular software to disseminate cancer statistics. Our goals were to improve upon the Tiwari [3] methods by including spatial autocorrelation and to develop a resulting method that could replace the current method in SEER*Stat. Therefore, we used a similar approach as Tiwari to develop our confidence interval and applied the new method to the same three cancer datasets so as to make the results most comparable.

Several limitations exist in this study. First, isotropy is assumed for spatial autocorrelation. It may be argued that direction as well as distance between regions will have an impact on disease rates. Considering anisotropy will greatly increase the complexity of model and computation. Secondly, the exponential semivariogram model that fit our lung cancer data well may not be the best choice for every outcome variable, although experts believe that the choice of semivariogram model is less important than the inclusion of spatial autocorrelation at all (see p. 379 of [10]).

We were limited in our ability to assess the impact of adding spatial autocorrelation to the rate ratio variance calculations for uncommon and rare cancers like esophagus and tongue cancers. Even when we simulated data with very strong spatial autocorrelation, the resulting data did not show much of a detectable spatial pattern. This is probably due to the small number of cases or the Modifiable Areal Unit Problem (MAUP). Assessing the impact of small numbers or MAUP to spatial autocorrelation is beyond the scope of this paper; our method was developed to account for spatial autocorrelation without regard to how it came about.

Another limitation of our approach is that we have underestimated the uncertainty of the rate ratio variance by assuming no error in the estimation of the covariance parameter estimates. It will be very important to explore approaches that fully account for the uncertainty in the spatial autocorrelation estimation in future work. However, because users are typically not interested in computing a rate ratio for areas with small populations, knowing that the confidence intervals will be large and uninformative in this situation, we suspect that uncertainty in the rate ratio estimate due to the estimation process will be small relative to uncertainty due to unmeasured spatial autocorrelation. Therefore, we believe that while we have shown that our method is superior to the Tiwari et al. method when spatial autocorrelation is present, and equivalent to it when area rates are uncorrelated, any further improvement resulting from a hierarchical (Bayesian) model that can compute the estimation process variation across many replicates will be relatively small. We have initiated a follow-up study to confirm this belief. It is impractical to implement a Bayesian estimation method that typically requires computation of thousands of replicates in a server- or web-based statistical system such as SEER*Stat, and therefore it is important to verify that the method proposed here, one that can easily be implemented, has captured nearly all of the variation in the rate ratio estimate.

## Conclusions

We have developed a method that takes into account spatial autocorrelation along with area overlap in calculating variances and confidence intervals of rate ratios between a sub-region and the total region. Our variance is comprised of three components, representing no correlation, area overlap, and spatial autocorrelation, respectively. We have shown that calculating the variance of the rate ratio including the possibility of spatial autocorrelation among the area-specific rates can lead to substantial improvements over the Tiwari method. For U.S. state-level cancer data, confidence intervals were shorter and power was greater than the Tiwari method. The Tiwari method accounted for correlation due to area overlap but not for spatial autocorrelation among the areas.

Application to simulated state-level data showed that the advantage of the proposed method is directly related to the strength and scale of the spatial autocorrelation. Improved results were also seen at the county level using simulated data based on population patterns in Kentucky counties. We did note two instances where results were not as good as in the Tiwari method, both where one region’s population constituted a large proportion of the population for the entire aggregated area (California compared to the U.S. and Jefferson County compared to Kentucky). In these cases, the correlation due to the population overlap was much larger than any observed spatial autocorrelation, eliminating any advantage for our method over methods that ignore this source of correlation. Because of the large population size in these regions, though, the variance and interval estimates were already precise, and adding spatial autocorrelation does not practically impact the interval estimates or the statistical power.

One approach to calculating confidence intervals for the rate ratio might be to assess the degree and scale of spatial autocorrelation among the areas and then decide on whether or not to include the spatial autocorrelation. However, these correlations are on a continuum, so it would be difficult to set a cutpoint beyond which the spatial method should be used. We have shown that our method provides the same results as the Tiwari method when there is very little spatial autocorrelation, and it does account for overlap between a sub-region and its parent region. Thus for little additional computational cost, we obtain an estimate equal to that currently used for SEER data if area-specific rates are independent, but in the presence of increasing spatial autocorrelation we can obtain substantial improvements over the existing method. Since the calculations are relatively easy to implement, we recommend using the new method in all cases.

## Abbreviation

CI: confidence interval
